# Plasma Circulating Tumor HPV DNA for the Surveillance of Cancer Recurrence in HPV-Associated Oropharyngeal Cancer

**DOI:** 10.1200/JCO.19.02444

**Published:** 2020-02-04

**Authors:** Bhishamjit S. Chera, Sunil Kumar, Colette Shen, Robert Amdur, Roi Dagan, Rebecca Green, Emily Goldman, Jared Weiss, Juneko Grilley-Olson, Shetal Patel, Adam Zanation, Trevor Hackman, Jeff Blumberg, Samip Patel, Brian Thorp, Mark Weissler, Wendell Yarbrough, Nathan Sheets, William Mendenhall, Xianming M. Tan, Gaorav P. Gupta

**Affiliations:** ^1^ Department of Radiation Oncology, University of North Carolina School of Medicine, Chapel Hill, NC; ^2^ Lineberger Comprehensive Cancer Center, University of North Carolina Hospitals, Chapel Hill, NC; ^3^ Department of Radiation Oncology, University of Florida Hospitals, Gainesville, FL; ^4^ University of North Carolina at Chapel Hill, Chapel Hill, NC; ^5^ Department of Medicine, Division of Hematology Oncology, University of North Carolina School of Medicine, Chapel Hill, NC; ^6^ Department of Otolaryngology/Head and Neck Surgery, University of North Carolina School of Medicine, Chapel Hill, NC; ^7^ Department of Pathology, University of North Carolina School of Medicine, Chapel Hill, NC; ^8^ Rex/UNC Hospital, Raleigh, NC

## Abstract

**PURPOSE:**

Plasma circulating tumor human papillomavirus DNA (ctHPVDNA) is a sensitive and specific biomarker of human papillomavirus (HPV)-associated oropharyngeal squamous cell carcinoma (OPSCC). We investigated whether longitudinal monitoring of ctHPVDNA during post-treatment surveillance could accurately detect clinical disease recurrence.

**METHODS AND MATERIALS:**

A prospective biomarker clinical trial was conducted among patients with nonmetastatic HPV-associated (p16-positive) OPSCC. All patients were treated with curative-intent chemoradiotherapy (CRT). Patients underwent a 3-month post-CRT positron emission tomography/computed tomography scan and were thereafter clinically evaluated every 2-4 months (years 1-2), then every 6 months (years 3-5). Chest imaging was performed every 6 months. Blood specimens were collected every 6-9 months for analysis of plasma ctHPVDNA using a multianalyte digital polymerase chain reaction assay. The primary endpoint was to estimate the negative predictive value (NPV) and positive predictive value (PPV) of ctHPVDNA surveillance.

**RESULTS:**

One hundred fifteen patients were enrolled, and 1,006 blood samples were analyzed. After a median follow-up time of 23 months (range, 6.1-54.7 months), 15 patients (13%) developed disease recurrence. Eighty-seven patients had undetectable ctHPVDNA at all post-treatment time points, and none developed recurrence (NPV, 100%; 95% CI, 96% to 100%). Twenty-eight patients developed a positive ctHPVDNA during post-treatment surveillance, 15 of whom were diagnosed with biopsy-proven recurrence. Sixteen patients had 2 consecutively positive ctHPVDNA blood tests, 15 of whom developed biopsy-proven recurrence. Two consecutively positive ctHPVDNA blood tests had a PPV of 94% (95% CI, 70% to 99%). Median lead time between ctHPVDNA positivity and biopsy-proven recurrence was 3.9 months (range, 0.37-12.9 months).

**CONCLUSION:**

Detection of ctHPVDNA in two consecutive plasma samples during post-treatment surveillance has high PPV and NPV for identifying disease recurrence in patients with HPV-associated oropharyngeal cancer and may facilitate earlier initiation of salvage therapy.

## INTRODUCTION

High-risk strains of the human papillomavirus (HPV) are major causative agents of oropharyngeal, cervical, vulvar, vaginal, and anal squamous cell cancers. The Centers for Disease Control and Prevention estimates that approximately 42,700 new cases of HPV-associated cancers occur in the United States each year.^1^ Over the past decade, oropharyngeal squamous cell carcinoma (OPSCC) has become the most prevalent HPV-associated cancer in the United States, and the incidence is rapidly increasing year by year.^2,3^ The treatment outcomes for HPV-associated OPSCC are more favorable than HPV-negative OPSCC.^4^ Consequently, efforts to de-intensify therapy for HPV-associated OPSCC are actively being pursued to reduce treatment-related toxicities.^5-10^ However, approximately 10%-25% of patients will develop disease recurrence depending on clinical risk factors and tumor biology.

Whereas most recurrences of OPSCC occur within the first 2 years of post-treatment surveillance, HPV-associated OPSCC can recur up to 5 years after treatment, and rare case reports have described even longer latency periods.^11,12^ Although distant recurrence of HPV-associated OPSCC is most commonly observed in the lungs, recurrences can also occur in atypical sites (eg, liver, bones, and brain)^11,13^. Despite these unpredictable patterns of relapse, recurrent/metastatic HPV-associated OPSCC has significantly better survival outcomes after salvage therapy than HPV-negative OPSCC.^14,15^

After definitive treatment, a 3-month positron emission tomography/computed tomography (PET/CT) scan is standardly performed to assess response.^16^ National Comprehensive Cancer Network (NCCN) guidelines for surveillance of patients with HPV-associated OPSCC are clinical examinations every 1 to 3 months in year 1, every 2 to 6 months in year 2, every 4 to 8 months in years 3 to 5, and then once a year thereafter.^17^ Often, an in-office fiberoptic nasopharyngolaryngoscopy is performed with each visit, although a recent study has shown that routine clinical surveillance rarely identifies disease recurrence.^18^ Post-treatment imaging of the neck and chest can be considered at 6 months and yearly thereafter (category 2B NCCN consensus).^17^

A blood-based surveillance test has the potential to facilitate early detection of cancer recurrence, as has recently been demonstrated for bladder, breast, and colorectal cancers using personalized circulating tumor DNA assays.^19-22^ Circulating tumor HPV DNA (ctHPVDNA) has emerged as a promising biomarker for HPV-associated OPSCC, because approximately 90% of patients have detectable plasma ctHPVDNA at the time of diagnosis.^23-25^ Dynamic changes in ctHPVDNA levels correlate with treatment response in patients with either localized or metastatic HPV-associated OPSCC.^23,26^ The clinical utility of longitudinal ctHPVDNA monitoring for surveillance of cancer recurrence after curative-intent therapy has not been established.

We describe results from a prospective biomarker study of longitudinal ctHPVDNA monitoring in a cohort of patients with HPV-associated OPSCC who had no clinical evidence of disease after definitive chemoradiotherapy (CRT). We analyzed ctHPVDNA using a previously validated multianalyte digital polymerase chain reaction (PCR) assay that detects the 5 most common high-risk HPV strains associated with OPSCC (16, 18, 31, 33, and 35).^23^ Our hypotheses were that ctHPVDNA-based surveillance would have excellent negative predictive value (NPV) and positive predictive value (PPV) and may facilitate earlier detection of recurrence relative to routine clinical follow-up.

## METHODS AND MATERIALS

### Study Design, Eligibility/Patient Sample Collection, and Clinical Data Abstraction

All patients included in this study provided written informed consent to an institutional review board (IRB)-approved prospective biomarker study at the University of North Carolina at Chapel Hill, University of Florida at Gainesville and Jacksonville, and REX/UNC Hospital in Raleigh, NC. Patients with HPV-associated OPSCC were enrolled in an IRB-approved prospective biomarker study (ClinicalTrials.gov identifier: NCT0316182) and/or in 1 of 2 institutional, single-arm, phase II, de-intensified CRT trials (ClinicalTrials.gov identifier: NCT02281955 or NCT03077243). The major eligibility criteria were (1) 18 years of age or older; (2) biopsy-proven OPSCC; (3) p16 positivity (defined as > 70% of carcinoma cells showing nuclear reactivity); (4) T0 to T4, N0 to N3, M0 (American Joint Committee on Cancer [AJCC] 8th edition); and (5) receiving definitive CRT.

### Definitive CRT and Post-Treatment Surveillance

Patients not co-enrolled in the phase II de-intensification CRT trials received standard-of-care 70 Gy intensity-modulated radiotherapy (IMRT) with concurrent chemotherapy (eg, cisplatin 100 mg/m^2^ × 3 cycles). All patients enrolled in the phase II de-intensification CRT trials received 60 Gy IMRT with concurrent weekly intravenous cisplatin 30 mg/m^2^. Patients with T0-T2 N0-1 (AJCC 7th edition) disease did not receive chemotherapy. All patients underwent a 3-month post-treatment PET/CT to determine the need for planned neck dissection. Thereafter, patients underwent clinical examinations every 2-4 months for years 1-2, then every 6 months for years 3-5. Chest imaging was performed every 6 months. De-identified blood specimens were collected pretreatment, weekly during CRT, and with each post-treatment follow-up visit for ctHPVDNA analysis. Determination of disease recurrence for this study required tissue biopsy confirmation.

### ctHPVDNA Analyses

Blood samples were collected in 10-mL cell-free DNA BCT blood collection tubes (Streck, La Vista, NE; catalog No. 218962), and double-spun plasma (2,000×g) was harvested within 3 days of collection. Plasma cell-free DNA (cfDNA) was extracted from 2-5 mL of plasma using the QIAamp circulating nucleic acid kit (Qiagen, Valencia, CA; catalog No. 55114). The amount of purified cfDNA was quantified with a Qubit fluorometer and PicoGreen quantification reagents (Invitrogen, Carlsbad, CA).

Validated digital PCR (dPCR) assays were performed on the QX-200 platform (Bio-Rad, Hercules, CA), and analyzed using QuantaSoft software v1.7.4.0917 (Bio-Rad). Sample quality was assessed by performing a dPCR assay targeting a region of the *ESR1* gene on chromosome 6 as previously described.^23^ Primers and 5′ hydrolysis probes were designed to specifically detect amplicons within the E6 and E7 genes encoded by high-risk HPV strain 16 and E7 gene for high-risk HPV strains 18, 31, 33, and 35, details of which have been previously described.^23^ This previously validated ctHPVDNA assay distinguishes tumor-derived fragmented HPV DNA from native HPV genomic DNA, regardless of tumor HPV integration status.^23^ Test positivity cutoffs were predefined and prospectively applied to clinical specimens. Study investigators who were responsible for determining ctHPVDNA status were blinded to the patient’s clinical status.

### Statistical Analyses

The primary objective of this study was to estimate the NPV and PPV for ctHPVDNA-based surveillance to identify patients with recurrence of HPV-associated OPSCC. We reasoned that a blood-based surveillance test would be useful if the NPV was > 90%. With a sample size of 115 patients and an estimated 2-year recurrence rate of approximately 12%,^8^ we anticipated that approximately 100 patients would test negative for the blood test. This sample size yielded 80% power to claim the test was useful (ie, NPV > 90%) at a 1-sided alpha level .05, if the true NPV was 97% or higher. Recurrence was defined as biopsy-proven disease that was diagnosed after the 3-month post-treatment PET/CT. Kaplan-Meier estimates of local control (LC), regional control (RC), local-regional control (LRC), distant metastasis–free survival (DMFS), relapse-free survival (RFS), cause-specific survival (CSS), and overall survival (OS) were calculated relative to the last day of CRT. Statistical significance was assessed using a 2-tailed log-rank test. Ninety-five percent binomial CIs were calculated using the Clopper-Pearson method.

## RESULTS

### Patient Cohorts, Clinical Characteristics, and Cancer Control Outcomes

**FIG 1.**
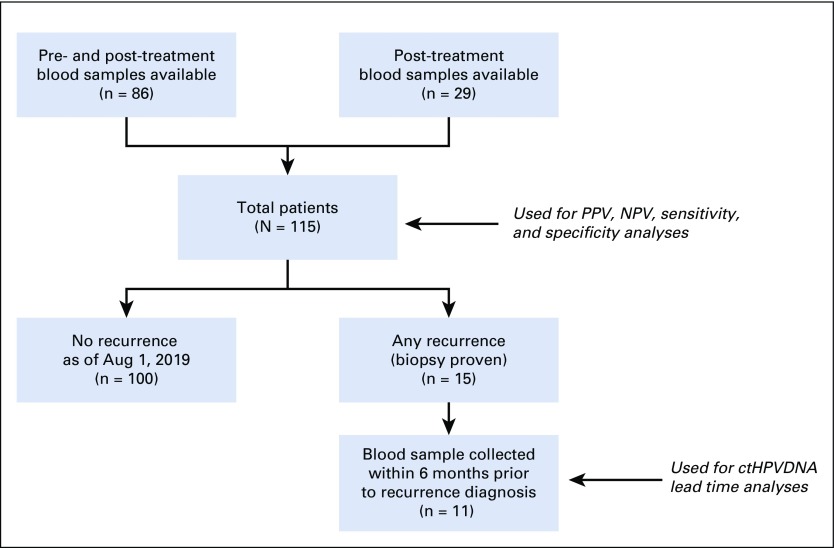
REMARK diagram of study population and patient subsets used for study analyses. ctHPVDNA, circulating tumor human papillomavirus DNA; NPV, negative predictive value; PPV, positive predictive value.

Between March 2016 and August 2018, 115 patients with stage I to III HPV-associated OPSCC were enrolled. The clinical characteristics of the study population are shown in Table 1. A total of 97 patients (84%) received de-intensified CRT in the clinical trial (60 Gy). Eighty-six of 115 patients had pre-CRT and during-CRT blood samples available; post-treatment blood samples were analyzed for all patients (Fig 1).

**TABLE 1.**
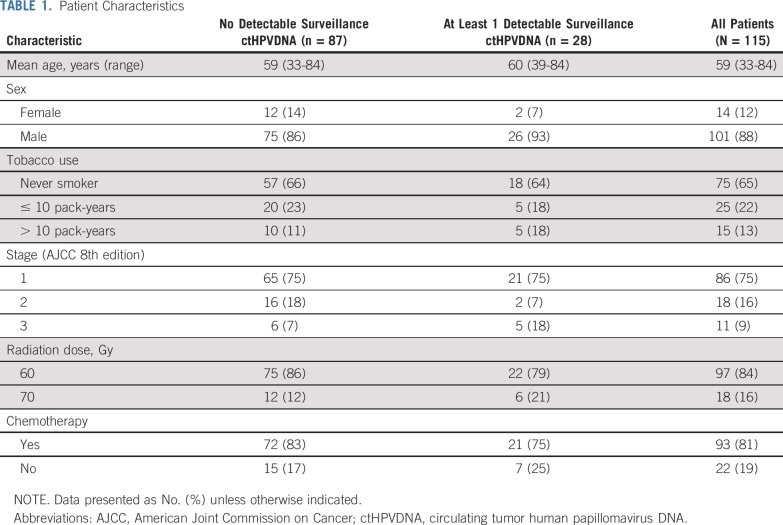
Patient Characteristics

The median follow-up was 23.7 months (range, 6.1-54.7 months). Fifteen patients had a recurrence: 1 local only, 1 regional only, 10 distant only, 1 local and distant, and 2 regional and distant. The actuarial 2-year estimates of LC, RC, LRC, DMFS, RFS, CSS, and OS were 98%, 97%, 96%, 86%, 83%, 97%, and 97%, respectively.

### Longitudinal ctHPVDNA Monitoring

**FIG 2.**
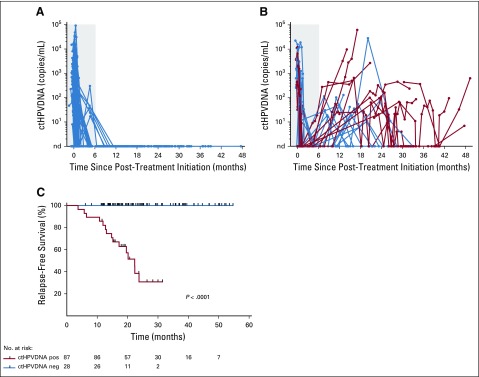
Longitudinal ctHPVDNA surveillance identifies patients at high risk of disease recurrence. (A) Eighty-seven out of 115 patients (76%) had undetectable ctHPVDNA levels at all post-treatment surveillance time points (starting at 6 months after start of CRT). (B) Twenty-eight patients had a positive ctHPVDNA test result during post-treatment surveillance. Red lines indicate patients who have been diagnosed with biopsy-proven disease recurrence. The blue shaded region indicates the interval for initial treatment with CRT and assessment of response by positron emission tomography/computed tomography, which was not included in the post-treatment surveillance period. (C) Kaplan-Meier relapse-free survival of patients with undetectable ctHPVDNA at all surveillance time points versus patients with at least one abnormal ctHPVDNA blood test. *P* value was calculated using a two-tailed log-rank test. ctHPVDNA, circulating tumor human papillomavirus DNA; neg, negative; pos, positive.

A total of 1,006 blood samples from 115 patients were collected, 999 of which were evaluable for ctHPVDNA (99.3% assay success rate). Eighty-seven of 115 patients (76%) had undetectable ctHPVDNA at all follow-up time points (Fig 2A). None of these patients had been diagnosed with recurrent disease, corresponding to an NPV of 100%. Twenty-eight patients had an abnormal ctHPVDNA signal during surveillance (Fig 2B). Median time to abnormal ctHPVDNA signal was 12.3 months after completing CRT (range, 2.6-29.1 months). Median ctHPVDNA level at the time of the first abnormal blood test was 71 copies/mL plasma (range, 7-244,615 copies/mL). Fifteen of these patients have been diagnosed with disease recurrence as of August 1, 2019, for a PPV of 54% (95% CI, 0.339 to 0.725). Actuarial 2-year RFS rates were 30% for patients with an abnormal ctHPVDNA blood test during post-treatment surveillance and 100% for all remaining patients (*P* < .0001; Fig 2C).

### Spontaneous Clearance of Abnormal ctHPVDNA in a Subset of Patients

**FIG 3.**
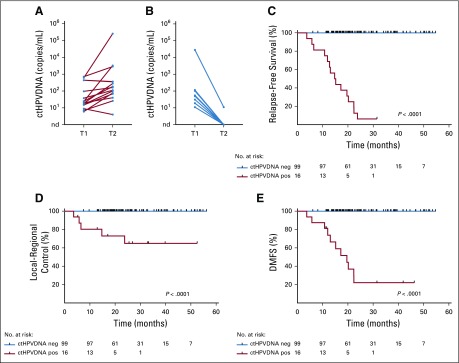
Patients with two consecutively abnormal ctHPVDNA surveillance tests have a higher risk for disease recurrence. ctHPVDNA levels at the first abnormal surveillance time point (T1) and a subsequent time point (T2) for patients with (A) biopsy-proven disease recurrence or (B) no clinically evident disease recurrence. Kaplan-Meier estimates for (C) relapse-free survival, (D) locoregional control, and (E) distant metastasis-free survival for patients who had two consecutively abnormal ctHPVDNA surveillance tests (ctHPVDNA positive) versus those without two consecutively abnormal ctHPVDNA surveillance tests (ctHPVDNA negative). *P* values were calculated using a two-tailed log-rank test. ctHPVDNA, circulating tumor human papillomavirus DNA; neg, negative; pos, positive.

We analyzed ctHPVDNA kinetics more closely in a subset of 24 patients in whom a follow-up blood test was available after an initially positive ctHPVDNA test during post-treatment surveillance. Among the 15 patients who developed recurrence, ctHPVDNA levels remained elevated at a subsequent time point in all patients (Fig 3A). In contrast, 8 of 9 patients who did not develop disease recurrence had clearance of ctHPVDNA by the subsequent blood collection time point (Fig 3B). One patient had low levels of residual ctHPVDNA (< 1% of the peak value) that became undetectable at the following time point. These analyses indicate that patients who develop recurrent or metastatic disease have persistently elevated ctHPVDNA, whereas approximately 8% of patients in our cohort developed a transient elevation in ctHPVDNA during post-treatment surveillance that spontaneously resolved without intervening treatment and without any clinical evidence of recurrent disease.

### Detection of Recurrence With Two Consecutively Abnormal ctHPVDNA Levels

Based on the observation that some patients develop a transient spike in ctHPVDNA without later developing recurrence, we performed a separate analysis considering two consecutively abnormal ctHPVDNA tests as a criterion for identifying patients at highest risk for relapse. Using this classification, 99 of 115 patients in our study (86%) had negative ctHPVDNA surveillance, and none of these patients developed recurrence (NPV, 100%; 95% CI, 96% to 100%). In contrast, 15 of 16 patients with abnormal ctHPVDNA surveillance developed biopsy-proven recurrence (PPV, 94%; 95% CI, 70% to 100%). Sensitivity and specificity were 100% and 99%, respectively. Actuarial 2-year RFS was 5% in patients with two consecutively abnormal ctHPVDNA tests, whereas it was 100% in all remaining patients (Fig 3C; *P* < .0001). Two-year LRC and DMFS among patients with two consecutively abnormal ctHPVDNA tests were 65% and 21%, respectively (Figs 3D and 3E), compared with 100% for the remaining 99 patients in the study cohort.

### Early Detection of Recurrence With ctHPVDNA

**FIG 4.**
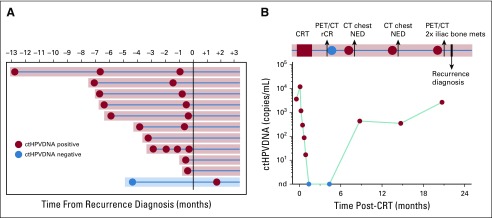
ctHPVDNA surveillance facilitates early detection of disease recurrence. (A) Modified swimmer plot illustrating positive and negative ctHPVDNA surveillance tests in 11 patients with recurrence who had a blood sample available for analysis within 6 months prior to biopsy-proven recurrence. Day 0 is day of biopsy proven recurrence. Radiographic detection of recurrence occurred approximately 2 weeks prior to day 0. Ten out of 11 patients had an abnormal ctHPVDNA test result prior to recurrence diagnosis (shaded in red). (B) ctHPVDNA kinetics and clinical surveillance imaging results for a study patient with a 14-month interval from the first abnormal ctHPVDNA blood test result and clinical diagnosis of disease recurrence. CRT, chemoradiotherapy; CT, computed tomography; ctHPVDNA, circulating tumor human papillomavirus DNA; mets, metastases; NED, no evidence of disease; PET, positron emission tomography; rCR, radiographic complete response.

A blood draw was obtained within 6 months before biopsy-proven recurrence in 11 of 15 patients. In 10 of these 11 patients, abnormal ctHPVDNA was detected before recurrence diagnosis with routine clinical follow-up (Fig 4A). One patient had undetectable ctHPVDNA 3.5 months before recurrence, but it was detectable at the next available blood specimen collection, approximately 1.5 months after recurrence diagnosis. Median lead time from the first abnormal ctHPVDNA blood test to biopsy-proven recurrence was 3.9 months (range, 0.37-12.9 months). There were four instances of radiographic examinations that failed to identify recurrence in patients with abnormal ctHPVDNA who were later diagnosed with biopsy-proven recurrence. In each of these patients, the site of recurrence was outside of the body regions being imaged. One patient demonstrated a 14-month lead time by ctHPVDNA (Fig 4B). This patient presented with T2N1(AJCC 8th edition) HPV-associated oropharyngeal cancer and achieved a radiographic complete response by PET/CT 3 months after completing CRT, with a corresponding clearance of ctHPVDNA levels in plasma. The patient developed ctHPVDNA recurrence 3 months later, which was persistently elevated for two additional blood sampling time points. During this time, the patient underwent CT scans of the head, neck, and chest that did not identify recurrent disease. After the third abnormal ctHPVDNA test result, the patient underwent a PET/CT that revealed 2 large metastases in the iliac bone.

### Patterns of Disease Recurrence

**FIG A1.**
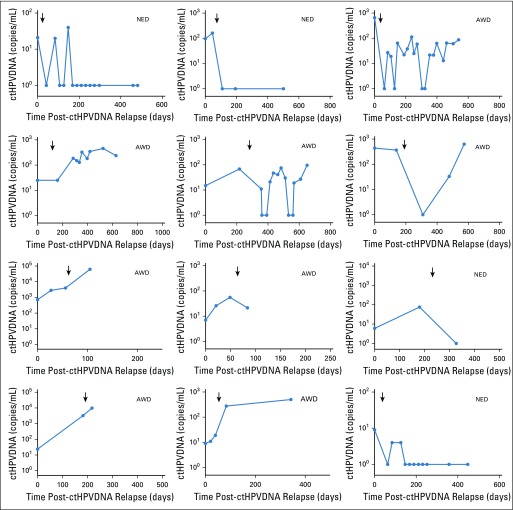
ctHPVDNA kinetics after salvage therapy in patients with recurrence. ctHPVDNA kinetics or 12 patients with clinical recurrence who had blood samples analyzed after initiation of salvage therapy (indicated by arrow). Current disease status is also shown for each plot, and corresponds to residual ctHPVDNA levels. AWD, alive with disease; ctHPVDNA, circulating tumor human papillomavirus DNA; NED, no evidence of disease.

Disease recurrence was predominantly asymptomatic (14/15; 93%). Clinical examinations did not identify recurrence in any of these 14 patients, consistent with another recent study.^18^ All recurrences were initially confined to a single organ system, and 10 patients (67%) presented with oligorecurrence (≤ 3 lesions). Surgery and/or salvage radiotherapy was used in 8 patients (53%). Seven patients (47%) received immune checkpoint inhibitor therapy. As previously reported, ctHPVDNA levels responded to salvage therapy and correlated with disease control (Fig A1). As of August 1, 2019, the clinical status of the 15 patients with recurrence was as follows: 3 patients died as a result of disease, 7 patients were alive with disease, and 5 patients had no evidence of disease.

## DISCUSSION

HPV-associated OPSCCs recur later, have a higher prevalence of metastases outside of the lungs, and are associated with a longer postrecurrence survival than HPV-negative OPSCC. Patients who are identified with recurrence earlier and with fewer sites of recurrence have longer postrecurrence survival.^15,18^ Clinical and radiographic surveillance must balance cost versus benefit from early detection of recurrent disease and thus is heterogeneously offered to patients. Our study demonstrates that ctHPVDNA monitoring has excellent sensitivity (100%) and specificity (99%) for identifying patients with clinical recurrence and thus represents an attractive strategy to improve the effectiveness and cost-efficiency of post-treatment surveillance paradigms for HPV-associated OPSCC.

The NPV (100%) and PPV (94%) of recurrence detection by ctHPVDNA compares favorably with alternative and emerging post-treatment surveillance strategies. PET/CT has been shown to have high NPV (100%) for recurrence detection, but can yield false-positive results that limits the PPV (77%).^27^ A recent prospective biomarker study investigated oral HPV DNA testing during post-treatment surveillance and reported NPV 86% and PPV 43% for recurrence detection.^28^ The sensitivity for oral HPV DNA persistence to identify distant recurrence was only 27%. Comparatively, plasma ctHPVDNA surveillance had 100% sensitivity for detecting both local-regional and distant recurrences in our cohort, suggesting that plasma is the preferred site for monitoring HPV DNA in the post-treatment setting. Our findings are similar to other recent studies of personalized ctDNA monitoring after curative-intent therapy for other cancer types.^19-22^ However, ctHPVDNA monitoring achieves equivalent or better sensitivity and specificity for recurrence detection without requiring the development of patient-specific genomic assays.

Approximately 75% of the patients in our study remained ctHPVDNA negative throughout surveillance, and none of these patients developed disease recurrence (NPV, 100%). Thus, ctHPVDNA surveillance could be used to select patients for whom radiographic imaging tests may be spared. Omission of additional scans beyond the 3-month post-treatment PET in these patients would reduce health care costs and mitigate the financial toxicity of cancer survivorship.^29^ In-office nasopharyngolaryngoscopy may also be omitted based on recent evidence of minimal clinical utility.^18^ A potential impact of ctHPVDNA-based surveillance on patient quality of life should be evaluated in future studies.

An unexpected finding of our study was the observation that a subset of patients (< 10%) experienced a transient spike in ctHPVDNA that spontaneously resolved without intervening treatment or development of disease recurrence. It is unlikely that these patients represent technical false positives of the ctHPVDNA assay, because each of these samples was confirmed positive in three replicates, and the analytical false-positive rate of the assay was < 1%. An intriguing possibility is that these patients may have experienced subclinical recurrence that was immunologically cleared. Additional studies of ctHPVDNA kinetics and correlation with immune-related biomarkers are warranted for this subset of patients.

Use of two consecutively positive ctHPVDNA tests as a criterion for identifying patients with a high risk of recurrent disease had a PPV of 94%. Increases in ctHPVDNA preceded biopsy-proven recurrence in the majority of patients, with a median of approximately 3.9 months. Based on these findings, increasing the frequency of ctHPVDNA testing to every three months may be more effective than the 6-month interval used in this study. Interestingly, we did not observe any instances of molecular relapse that were not visualized by PET/CT imaging. There were four instances of “missed” disease recurrence when ctHPVDNA was abnormal and CT-based surveillance imaging did not include the site of recurrence. Thus, use of whole-body PET/CT scans in patients with two consecutively positive ctHPVDNA tests may be the most cost-effective strategy for identifying disease recurrence. ctHPVDNA may also confirm recurrent disease when tissue biopsy is deemed high risk.

Whether earlier detection of disease recurrence may positively impact survival outcomes in HPV-associated OPSCC remains an open question. ctHPVDNA-based monitoring may result in a higher rate of identifying oligorecurrence and potentially a higher rate of salvage with surgery or radiotherapy. Thus, clinical trials of early intervention after ctHPVDNA-based detection are warranted.

The findings described here are specific for the optimized multianalyte ctHPVDNA assay used in this study and may not apply to alternative ctHPVDNA assays. Furthermore, detection of two consecutively positive ctHPVDNA tests to improve the PPV was not prospectively defined and warrants further validation. Consistent with NCCN guidelines, there is no “gold standard” for surveillance imaging. Thus, we opted to use a more stringent criterion of biopsy-proven recurrence to define a change in disease status. A shorter interval between ctHPVDNA testing (eg, every 3 months) would more precisely define the extent of earlier detection. Several heterogenous clinical factors (intensity of treatment, tobacco pack-years, use of chemotherapy) may have impacted the pattern and frequency of disease recurrence in this study. Although our study demonstrates feasibility of ctHPVDNA-based surveillance for both distant and local-regional recurrences, additional investigation is needed to explore potential differences in detection efficiency. In summary, post-treatment surveillance of cancer recurrence with plasma ctHPVDNA monitoring has exceptional NPV (100%) and PPV (94%) when using two consecutively positive ctHPVDNA tests as the criterion for positivity. Patients who have undetectable ctHPVDNA during clinical follow-up are unlikely to have recurrent disease and may be spared routine radiographic and in-office nasopharyngolaryngoscopic surveillance. Future studies should explore whether ctHPVDNA-based monitoring may help to reduce the financial toxicity of cancer survivorship and whether earlier detection of cancer relapse improves postrecurrence survival outcomes.
